# Fulminant Eye Infection in a Patient With Nephrotic Syndrome: A Case Report

**DOI:** 10.7759/cureus.55842

**Published:** 2024-03-09

**Authors:** Aditi S Kulkarni, Archana R Thool, Sachin Daigavane

**Affiliations:** 1 Ophthalmology, Jawaharlal Nehru Medical College, Datta Meghe Institute of Higher Education and Research, Wardha, IND

**Keywords:** pseudomonas infections, corneal perforation, nephrotic syndrome, immunosuppression, endophthalmitis

## Abstract

This case report presents the clinical course of a 53-year-old male farmer with nephrotic syndrome, specifically focal segmental glomerulosclerosis, who developed a fulminant eye infection. While receiving maintenance hemodialysis and immunosuppressive therapy, the patient presented with sudden onset redness, discharge, and decreased vision in his right eye. Initial management with topical antibiotics and steroids failed to halt the progression of the infection, leading to corneal perforation and iris prolapse within a few days. Despite the discontinuation of immunosuppressive medications and initiation of broad-spectrum antimicrobial therapy, the patient's compromised renal function and anaemia precluded surgical intervention. This case underscores the challenges in managing severe ocular infections in immunocompromised patients. It highlights the importance of early recognition, aggressive antimicrobial therapy, and close ophthalmologic monitoring in preventing sight-threatening complications. Despite intensive management, the prognosis for visual recovery in such cases may be poor, emphasizing the need for preventive strategies and careful surveillance in high-risk patient populations.

## Introduction

Nephrotic syndrome, characterized by proteinuria, hypoalbuminemia, edema, and hyperlipidemia, is a heterogeneous group of glomerular diseases with varying etiologies and clinical presentations [1]. Focal segmental glomerulosclerosis (FSGS) is one of the primary histologic patterns observed in nephrotic syndrome, characterized by sclerosis affecting some but not all glomeruli [2]. Patients with nephrotic syndrome are at an increased risk of infections due to alterations in immune function, including impaired T cell-mediated immunity, complement dysregulation, and loss of immunoglobulins in the urine [3]. Furthermore, immunosuppressive medications, such as corticosteroids and calcineurin inhibitors, further predispose these individuals to infections [4].

Hemodialysis, a common treatment modality for end-stage renal disease, further compromises immune function by inducing leukopenia, impairing neutrophil function, and altering cytokine profiles [5]. Consequently, hemodialysis patients are particularly susceptible to infections affecting the ocular structures. Ocular infections in immunocompromised patients can present atypical features and may progress rapidly, leading to severe complications such as corneal perforation and endophthalmitis [6]. Prompt recognition and management of these infections are crucial to prevent irreversible visual impairment and systemic spread of infection. In this context, we present a case of fulminant eye infection in a patient with nephrotic syndrome, specifically FSGS, undergoing maintenance hemodialysis, highlighting the challenges in diagnosing and managing ocular complications in immunocompromised individuals.

## Case presentation

**Figure 1:**
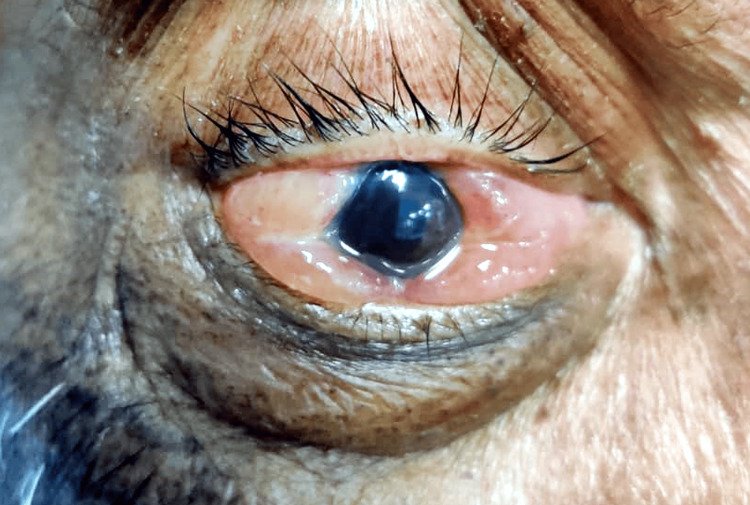
Displaying significant chemosis, eyelid edema, and congestion upon admission to the intensive care unit

A 53-year-old male farmer, previously undergoing treatment for nephrotic syndrome (specifically FSGS), was admitted to the intensive care unit due to complications with the dialysis. He had been on maintenance hemodialysis for the past six months. He presented with sudden onset redness and discharge in his right eye Figure 1, which progressively worsened without pain. Accompanied by severe chemosis and a decrease in vision, he experienced difficulty in opening his eyelid. Examination under a torchlight revealed a clear cornea in the right eye, with sluggish pupil reaction and evidence of cataractous changes in the lens. Due to dense cataract alterations, a fundus examination was not feasible. Visual acuity in the right eye was limited to light perception. Initial treatment comprised moxifloxacin (0.5% w/v) eye drops once daily and atropine (1% w/v) three times a day as a topical therapy. An MRI of the brain and orbit was recommended to rule out orbital cellulitis.

**Figure 2:**
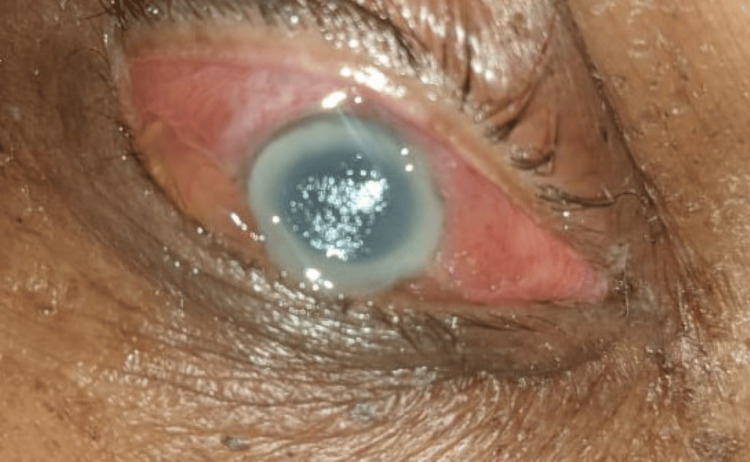
Exhibiting widespread corneal edema with a ring abscess located posterior to the cornea, accompanied by conjunctival congestion on the second day of admission to the intensive care unit

Within a day, he exhibited diffuse haziness of the cornea and peripheral corneal whitening. A ring abscess was suspected, primarily affecting the posterior cornea, although corneal scraping could not be obtained Figure 2. Notably, there was no corneal epithelial defect, and visual acuity remained limited to light perception in the right eye.

**Figure 3:**
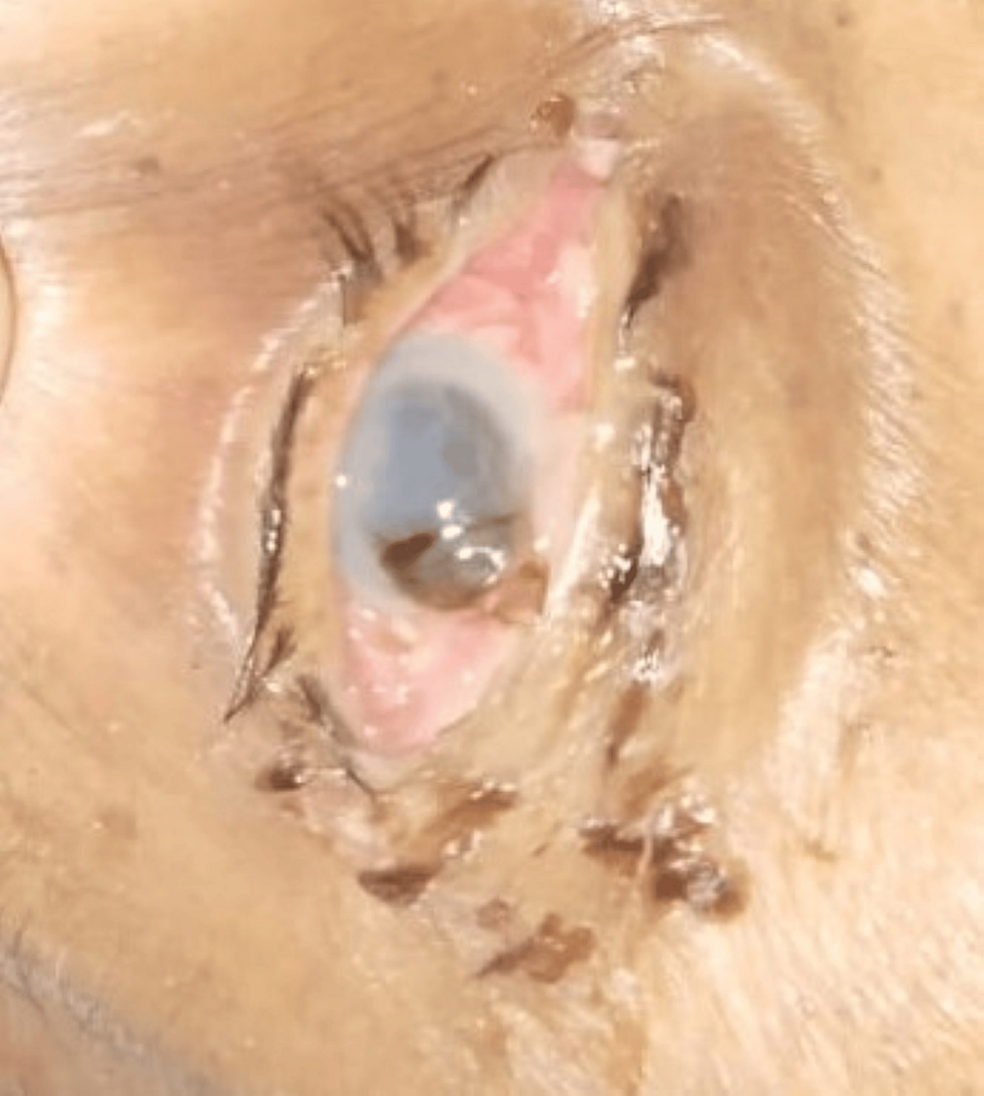
Manifested as corneal perforation with iris prolapse on the fourth day of admission to the intensive care unit

By day four, the patient manifested corneal perforation accompanied by iris prolapse Figure 3, coinciding with a loss of consciousness, rendering visual acuity assessment unfeasible. Examination of the left eye revealed normal findings. The patient had been receiving intravenous Tigecycline and oral steroids (Prednisolone 50 mg/day), which were discontinued on day two of examination upon observation of the ring abscess. MRI reports indicated no abnormalities in the brain or orbit. Although therapeutic penetrating keratoplasty was recommended post-perforation, the patient was deemed unfit for surgery by the anesthesiologist. Continuing topical treatment with eye patching was advised until stabilization. Further investigations revealed deranged renal function with anemia, while other biochemical and cardiac evaluations yielded unremarkable results.

## Discussion

The presented case underscores the challenges in managing fulminant eye infections in immunocompromised patients, particularly those with nephrotic syndrome undergoing maintenance hemodialysis. Despite prompt initiation of antimicrobial therapy and cessation of immunosuppressive medications, the infection rapidly progressed, resulting in corneal perforation and iris prolapse, ultimately compromising visual acuity and necessitating supportive measures. Patients with nephrotic syndrome are predisposed to infections due to immune dysregulation, proteinuria, and the use of immunosuppressive therapies [7]. Hemodialysis further compromises immune function, increasing susceptibility to infections [8]. Consequently, ocular infections in these individuals may manifest atypically and progress rapidly, as observed in the presented case.

The etiology of the eye infection in this case remains uncertain. While bacterial infections are common in patients with corneal ulcers, the absence of corneal epithelial defects and rapid progression of symptoms raise suspicion for atypical pathogens, such as fungi or viruses [6]. However, the exact causative organism could not be identified due to the inability to obtain corneal scrapings for microbiological analysis. Management of fulminant eye infections in immunocompromised patients poses significant challenges. Prompt initiation of broad-spectrum antimicrobial therapy is crucial to mitigate further damage and prevent systemic spread of infection [9]. In this case, systemic antibiotics were administered, but the infection progressed rapidly despite therapy.

The decision to forego therapeutic penetrating keratoplasty highlights the complexities involved in managing critically ill patients with multiple comorbidities. Although surgical intervention may offer the best chance for visual rehabilitation, it must be weighed against the patient's overall clinical status and risk of perioperative complications [10]. The long-term visual prognosis for the patient remains poor, given the extent of corneal damage and the potential for sequelae such as glaucoma or endophthalmitis. Close ophthalmologic monitoring and aggressive management of complications are essential to optimize outcomes in such cases.

## Conclusions

In conclusion, the presented case emphasizes the critical importance of prompt recognition and aggressive management of ocular infections in immunocompromised individuals, particularly those with nephrotic syndrome undergoing hemodialysis. Despite the administration of broad-spectrum antimicrobial therapy and cessation of immunosuppressive medications, the infection progressed rapidly, leading to severe corneal complications and significant visual impairment. This underscores the need for close collaboration between nephrologists and ophthalmologists to optimize patient outcomes and prevent irreversible ocular damage. Additionally, the decision-making process regarding surgical interventions such as penetrating keratoplasty should be carefully evaluated regarding the patient's overall clinical condition and comorbidities. Continuing research efforts are warranted to elucidate optimal management strategies for ocular complications in immunocompromised populations, with the ultimate goal of preserving visual function and improving the quality of life for these vulnerable individuals.
